# Colliding Epidemics and the Rise of Cryptococcosis

**DOI:** 10.3390/jof2010001

**Published:** 2015-12-23

**Authors:** Christina C. Chang, Sharon C.-A. Chen

**Affiliations:** 1 Department of Infectious Diseases, Alfred Hospital, Monash University, Melbourne 3181, Australia; christina.chang@monash.edu; 2 HIV Pathogenesis Programme, University of KwaZulu Natal, Durban 4001, South Africa; 3 Marie Bashir Institute for Emerging Infectious Diseases and Biosecurity, University of Sydney, Sydney 2145, Australia; 4 Centre for Infectious Diseases and Microbiology Laboratory Services, ICPMR—Pathology West, Westmead Hospital, Sydney 2145, Australia

**Keywords:** cryptococcosis, epidemiology, history, HIV, transplantation, outbreaks

## Abstract

Discovered more than 100 years ago as a human pathogen, the *Cryptococcus neoformans–Cryptococcus gattii* (*C. neoformans–C. gattii*) complex has seen a large global resurgence in its association with clinical disease in the last 30 years. First isolated in fermenting peach juice, and identified as a human pathogen in 1894 in a patient with bone lesions, this environmental pathogen has now found niches in soil, trees, birds, and domestic pets. Cryptococcosis is well recognized as an opportunistic infection and was first noted to be associated with reticuloendothelial cancers in the 1950s. Since then, advances in transplant immunology, medical science and surgical techniques have led to increasing numbers of solid organ transplantations (SOT) and hematological stem cell transplantations being performed, and the use of biological immunotherapeutics in increasingly high-risk and older individuals, have contributed to the further rise in cryptococcosis. Globally, however, the major driver for revivification of cryptococcosis is undoubtedly the HIV epidemic, particularly in Sub-Saharan Africa where access to care and antiretroviral therapy remains limited and advanced immunodeficiency, poverty and malnutrition remains the norm. As a zoonotic disease, environmental outbreaks of both human and animal cryptococcosis have been reported, possibly driven by climate change. This is best exemplified by the resurgence of *C. gattii* infection in Vancouver Island, Canada, and the Pacific Northwest of the United States since 1999. Here we describe how the colliding epidemics of HIV, transplantation and immunologics, climate change and migration have contributed to the rise of cryptococcosis.

## 1. Introduction

The history and biology of *Cryptococcus neoformans* (*C. neoformans*), now recognised as a species complex (the *Cryptococcus neoformans–Cryptococcus gatti* (*C. gattii*) complex), is detailed in an authoritative text by Casadavell and Perfect [1]. In the early 1990s, there were a smattering of reports of human and animal infection caused by *Cryptococcus*, including in the brain, lungs, skin and bone of humans, and of experimental infections in laboratory animals (reviewed in [1]). The introduction of corticosteroid therapy for various conditions and its association with such therapy with lymphoproliferative disorders stamped cryptococcosis as an opportunistic infection.

However, prior to 1981, cryptococcosis was a relatively rare condition (500–1000 cases a year in the United States (USA)) but with the onset of the HIV epidemic, 1200 cases of cryptococcosis were observed in New York City alone in 1991 [1]. Indeed, during this period, cryptococcal meningitis (CM) surpassed the combined numbers of all other causes of meningitis [1] and became the focus of intense research. The risks of cryptococcosis in patients who are immunosuppressed for reasons other than HIV/AIDS has also been of increasing interest. The spectrum of these patient groups is diverse, and includes patients with T-cell defects, hypogammaglobulinemia, transplant recipients and autoimmune conditions such as sarcoidosis and systemic lupus erythematosus. Prognosis of cryptococcosis was dismal in the early 1900s—with the exception of surgical removal of localized spinal lesions—until the antifungal agent, amphotericin B, was introduced in the late 1950s, which provided the first effective therapy against cryptococcosis. Today, both amphotericin B formulations and azole antifungals remain the mainstay of therapy.

## 2. Cryptococcosis in Association with Malignancy and Autoimmune Diseases

While the Scottish physician John Bennett and pathologist, Rudolph Virchow are often variously ascribed as the discoverers of leukemia, in fact the first description may be more accurately attributed to Peter Cullen in 1810, who described a 35 year old man with fevers and abdominal pain with milky blood, who he managed with “blood-letting” [2]. However, it was Bennett who in 1845 was the first to recognize that this “suppuration of the blood” was a primary disorder of the blood and later the condition was termed “*weisses blood*” and “leukemia” by Rudolph Virchow (reviewed in [2]). Hints of *Cryptococcus spp.* being an opportunistic pathogen occurring in patients with leukemia and other immunocompromised hosts arose with the first report of cryptococcosis (then cystic blastomyces or torulosis) in a patient with Hodgkin’s disease [3]. Interestingly, the authors inoculated guinea pigs with the spinal fluid collected from this patient intraperitoneally, intranasally and subcutaneously, and demonstrated this led to weight loss and death [3]. Cerebral infection with *Cryptococcus* was further demonstrated on autopsy after infection by the latter two routes [3].

In Gendel’s review of 165 patients with cryptococcosis (then torulosis), Hodgkin’s disease was coincident in 14 (8.5%) [4]. Of note, during this time, due to the propensity of cryptococci being recovered from enlarged lymph nodes, the notion of *Cryptococcus* as a cause of Hodgkin’s disease was posed [4]. An astute Dubin in 1947 suggested instead that a weakened immune system is a factor for the development of multiple concurrent infections in patients with Hodgkin’s disease [5]. In 1951, Collins *et al.* surmised a clear link between malignant reticulendothelial neoplasia (not only limited to Hodgkin’s disease) and cryptococcosis from his detailed review of 243 patients with cryptococcosis [6].

Kaplan *et al.* then followed with a comprehensive account of cryptococcal infection occurring in patients with neoplastic disease at Memorial Sloan Kettering Cancer Centre between 1956 and 1972 [7]. Forty-seven patients were diagnosed with cryptococcosis by culture or histopathology over 17 years, at an average of 2–4 cases a year [7]. Patients with chronic lymphocytic leukemia (CLL), Hodgkins’s disease, chronic myelocytic leukemia (CML) and multiple myeloma (MM) had the highest incidence of infection with rates of 24.3, 13.3, 10.9 and 6.9 per 1000 cases and, notably, only three patients had solid tumors [7]. Of the 23 patients who underwent autopsy, *Cryptococcus* was demonstrated in the central nervous system (CNS) in 20 patients, lungs—14, kidneys—six, spleen—five, lymph node—three, adrenals—two and one each of prostrate, thyroid, heart, pancreas and pericardium [7]. Of the 41 patients with neoplastic disease, 28 died within 60 days, despite amphotericin B being available to treat infection in the majority of cases [7]. Other infections include herpes zoster, tuberculosis (TB), nocardiosis, *Pneumocystis* and listeriosis, suggesting these patients were concurrently susceptible to intracellular pathogens [7].

Later, cryptococcosis became recognized as a common co-infection in multiple other immunodeficient states including autoimmune diseases and those with apparently normal immune status. These include the first cases of cryptococcosis in relation with sarcoidosis [8], with SLE [9], and in association with the use of steroids popularized in the 1950s [10,11]. Here, experiments using cryptococcal polysaccharide to stimulate white cells were also performed to refute *Cryptococcus* as the cause of these autoimmune phenomena [9]. Recently, a case of cryptococcosis was also reported in the setting of HyperIgM syndrome [9,12] and in a patient with a known “*Jak3*” gene mutation with severe combined immunodeficiency [13], raising an alert for physicians managing patients with primary immunodeficiencies.

## 3. The Rise of Transplantation and Transplant Immunology

Peter Medawar’s discovery of an immunological basis of graft rejection led us to the fundamental tenets of transplant immunology that a graft recipient reacts “against the graft” and will require immunosuppression for prolonged periods of time [14,15]. To this end, the ability of corticosteroid drugs to reduce rejection in animal models launched the idea of chemotherapeutic modulation of the human immune system (reviewed in [16]). Later, the development of purine and pyrimidine analogues including azathioprine and methotrexate [17] and cyclosporine, led to their successful use in cadaveric human renal transplants [18] and over the ensuing years, cyclosporine in particular, became the cornerstone of human transplantation. Since then, we have seen the development of newer drugs including Mycophenolate mofetil, tacrolimus [19] and Siroliumus [20], which have become key immunosuppressants in modern transplantation.

Surgical expertise has also contributed significantly to the pursuit for the perfect transplantation, including the first attempted kidney transplant in humans in 1906, the first successful renal transplantation (between identical twins) in 1954 [21], the first bone marrow transplant in 1957 [22,23], the first liver transplant in 1963 (reviewed in [24]), the first heart transplant in 1968 [22,23], the first heart–lung transplant in 1981 [22,23] and the first human allogeneic islet cell transplantation in 2000 [25]. Each patient group and transplant type mandates for lifelong, immunosuppression regimens with attendant lifelong risks for opportunistic infections including cryptococcosis. The looming possibility of porcine islet cell transplants from genetically-engineered pigs will bring new challenges [26]. The rapid progress in surgical techniques, organ preservation techniques, and newer effective immunosuppressants has contributed to an exponential growth in the number of transplantations. In 2012, the Global Observatory of Donation and Transplantation (GODT) reported at least 112,700 SOTS (a rise of 1.8% from 2011) with at least 77,800 renal transplants in a staggering 101 countries and at least 23,986 liver transplants in 68 countries [27]. These numbers are expected to rise with an expected rise in opportunistic infections.

This is well illustrated by the rising incidence of cryptococcosis in transplant recipients [27,28], largely driven by reactivation disease secondary to immunosuppression, though primary (*de novo*) infection via inhalation also occurs. Equally, donor-derived cryptococcosis has also been reported [29,30]. Of 52 SOT recipients with cryptococcosis described by Davis in 2009, organs transplanted included kidney (51.9%), liver (23.1%), lung (11.5%), heart (7.7.%), kidney–liver (1.9%), heart–lung (1.9%) and liver–pancreas–small bowel (1.9%) [28]. More recent reports of cryptococcosis post-SOT include those post-renal transplants [31,32,33], liver transplant [34], post-heart transplant [35], post-lung transplant [35,36] and a rare case of cryptococcosis post-autologous stem cell transplant [37]. While the majority present with CM, pulmonary infection and cutaneous cryptococcosis have also been reported [38,39].

Further, immune reconstitution inflammatory syndrome (IRIS) occurring in the setting of transplants is now a key area of research [40,41,42] with paradoxical cryptococcosis-associated immune reconstitution inflammatory syndrome (C-IRIS) occurring in ~14% of SOT-recipients who developed cryptococcosis at a median of 17 months post-transplant [41]. C-IRIS has been reported post kidney, liver, heart and kidney–liver transplant [41,42]. Patients with transplant-C-IRIS compared to those without C-IRIS, are more likely to have CNS involvement and disseminated disease at baseline and are associated with increased rates of discontinuation of calcineurin inhibitors [43].

## 4. Cryptococcosis and HIV

The HIV epidemic has undoubtedly been the greatest, most transformative challenge to global health in recent decades. Along with the first reports of Kaposi’s sarcoma in New York in 1981 and the discovery of the HIV virus in 1983 [17], CM became a common occurrence in American cities, to a point where it surpassed all other causes of meningitis combined. Recognized as an AIDS-defining illness (ADI) and often presenting as the first ADI, the lethality of advanced HIV and CM co-infection galvanized clinicians and researchers to focus on the delivery of antifungal therapy and management of raised intracranial pressure. Over the last 30 years, research in cryptococcosis–HIV co-infection has re-energized the field of cryptococcosis and *Cryptococcus* leading to nearly 10,000 research papers in this area of co-infection, and the recent creation of an international AIDS–Mycoses interest group.

In 2009, Park *et al.* reported an estimated million cases of CM occurring globally, of which two-thirds died within three months [44]. Not surprisingly, Sub-Saharan Africa bears >80% of these deaths [44], where the estimated number of deaths due to CM exceeds the number due to TB, and is behind only to malaria, diarrheal illnesses and childhood-cluster diseases [44]. More specifically, CM accounts for up to 20% of early deaths in HIV cohorts in Sub-Saharan Africa [45] and a history of CM is a strong predictor of post-antiretroviral therapy (ART) mortality [46].

A recent retrospective study of sera collected over the last 20 years showed a 2.9% prevalence rate of cryptococcal antigenemia in patients with advanced HIV in the U.S. (McKenney, *et al.* 2015, [47]). The arrival of ART stemmed the tide of HIV-related opportunistic infections, and epidemiological reports showed a clear reversal of the incidence of cryptococcosis in the United States in the early 2000s [48]. With the introduction of ART, the United States has seen a steady (5.8%) decline of cryptococcosis incidence, dropping to 1827 hospitalizations in 2009 nationwide [49]. The critical importance of ART in the management of HIV–CM co-infection is starkly illustrated in a paper from Zambia, where in an era without ART, all 230 patients with cryptococcosis died [50]. Distressingly, the median survival was only 19 days in those who received 200 mg of fluconazole daily and 10 days in those without access to any antifungal therapy [50]. All patients were dead at seven weeks when left untreated, and of those receiving fluconazole, none survived beyond 24 weeks [50].

Building on the pioneering work of John Bennett in the 1970s [50,51], van de Horst *et al.* performed a landmark trial in the era of HIV, published in 1997 [52] on which the current Infectious Diseases Society of America (IDSA) guidelines [53] are based. This multicenter, randomized, double-blinded trial studied 306 HIV-infected patients with CM and demonstrated that (i) the addition of 100 mg/kg/day 5-flucytosine (5-FC) to 1 mg/kg/day amphotericin B deoxycholate had higher mycological clearance at two weeks than amphotericin B deoxycholate alone and (ii) that fluconazole 200 mg daily was superior to Itraconazole 200 mg twice daily as consolidation therapy. Combination of amphotericin B and 5-FC for CM recently received the ultimate randomized controlled trial validation of conferring improved survival, in a large study of 299 patients [54].

Other smaller but equally important studies in HIV–CM co-infection over the last 20 years have been led by Thomas Harrison’s [55,56,57,58,59], David Boulware’s [60,61,62,63] and Peter Pappas’s [64] research groups based in Thailand, South Africa and Uganda, respectively. These clinical studies have compared antifungal therapy regimens and their route of administration [55,57], the role of adjuvant therapy [55,58,65], management of intracranial pressure [55,58,66,67] and the role of timing of ART commencement in the setting of active CM [62].

About 25%–30% of HIV–CM co-infected patients commencing ART develop C-IRIS [68,69] where patients represent with a flare of cryptococcal disease despite being on adequate ART and antifungal therapy. This is associated with repeated hospitalizations and increased morbidity and mortality. Increasing numbers of immunological studies in HIV–CM co-infection are being published as researchers attempt to understand the immunopathogenesis of HIV–CM co-infection and C-IRIS [61,70,71,72,73]. Just as HIV has challenged, yet also ironically strengthened, health systems globally, the scourge of HIV–CM co-infection has prompted research and care of CM in resource-limited settings which has, in turn, led to many insights applicable to other HIV co-infections.

All that said, in 2014, an estimated 36.9 million persons live with HIV globally of which 25.8 million reside in Sub-Saharan Africa [74]). Two million new HIV infections were reported in 2014 [74]. Overall, this equates to roughly 5600 new diagnoses per day, of which 600 occur in children [74]. While the last 30 years of HIV research has brought about much triumph, these figures highlight the continued challenge we face and the need for continued vigor and commitment to improving healthcare.

## 5. Environmental Outbreaks

Of more than 1.5 million species of fungi since their emergence 1.6 million years ago, only <0.02% are associated with human infection, of which only a few are thermo-tolerant (*i.e.*, able to grow at mammalian and higher temperatures) [75]. The ability of *Cryptococcus* spp. to grow optimally at human body temperatures (37 °C) is key to its virulence, and one of many reasons for its success as a human pathogen [76]. Intriguingly, transplant recipients from the southern United States have higher rates of cutaneous cryptococcosis compared to patients from other regions, even after controlling for confounders [39], though the mechanism for this remains unclear.

Climate change, global warming, natural disasters, pathogen evolution and shifts in host and vector populations may alter and pose new threats to the public [75,77,78]. The emergence of *C. gatti* in temperate climates, first coming to attention during the outbreak of *C. gattii* infections on Vancouver Island, British Columbia in 1999, is particularly illustrative. Previously thought to be restricted to tropical and subtropical climates, e.g., Central Africa, Cambodia, Thailand, Vietnam, Brazil, Australia, Hawaii and southern California [79], Stephen *et al.* [80], in 2002, reported a spike from the typical expectation of 4–6 cases a year, to 45 confirmed cases in animals and 50 in humans. Of the 45 infected animals, all but seven resided on the east coast of Vancouver Island and infected populations not only included cats and dogs, but also porpoises, ferrets and llamas [80]. Only three of the animals had received prior prednisolone [80].

This was followed by a comprehensive epidemiological study from 1999 to 2007 by Galanis *et al*. [81]. They reported a steady increase from six human cases per year in British Columbia in 1999, to 38 cases in 2006, totaling 218 cases, of which 73.9% resided on Vancouver Island and only 38% had an identifiable immunodeficiency [81]. While the incidence on Vancouver Island plateaued in 2002, the incidence on the North American mainland has increased [81]. Of note, *C. gattii* isolates of the molecular type (or genotype) VGII (subtype VGIIa and VGIIb) was found in 86.3% of confirmed cases [81] and recent coalescence gene genealogy analysis showed that this genotype is derived from ancient dispersal of *C. gattii* lineages originating from the Amazon rainforest [82]. From 2004 to 2010, 60 cases from the Pacific Northwest United States were reported to the Centre of Disease Control (CDC) of United States [83]. Following which, from 2009 to 2012, 25 human cases have been reported in eight non-Pacific Northwest states suggesting continued spread of this clonal strain with further subtyping studies delineating a novel subtype (VGIIc) not yet found outside the United States [84].

An appreciation of the distribution of molecular types by geographic region is central to the understanding of the current epidemiology of human infection, detailed here for *C. gattii* in Table 1. Genotyping of infective strains is particularly interesting in the setting of outbreaks and travel, both for tracking the origin of the isolate and for epidemiological purposes as illustrated by a Dutch returned traveler from Vancouver Island who acquired a *C. gattii* VGIIb infection [85]. Similarly, cryptococcal infections caused by molecular types VGIII and VGIV diagnosed in patients in Europe, have been elegantly linked to their prior exposure to regions in Africa [86].

Genotyping also allows recognition of strain evolution in the environment, illustrated by a novel local acquisition of VGIIa in a patient from Tokyo, where VGIIa had not previously been reported [87]. The impact of strain evolution, human travel, migration and environmental change in this already globalized world, on human and animal cryptococcosis will require continued, coordinated international epidemiological surveillance and response.

**Table 1:** Molecular types of *Cryptococcus gattii*: broad distribution by selected regions *.

Region	Genotype (% Total Strains)			
	VGI	VGII	VGIII	VGIV
North America	11	60	15	4
South America	12	69	15	4
Europe	70	30	-	-
Africa	11	1	2	86
Asia	73	19	5	3
Australasia	65	31	7	-

* Adapted from Chen *et al.* [88].

## 6. Conclusions

Progress in medical science particularly in the management of autoimmune illnesses, malignancy and transplantation, together with the ongoing burden and threat of HIV globally and the imminent peril of climate change and environmental outbreaks have combined to chart the history and rise of cryptococcosis over the last 100 years. Sadly, 100 years later, mortality rates remain unacceptably high, particularly in resource-limited settings. Earlier diagnosis and treatment of cryptococcosis, newer antifungal therapy options and a better understanding of host–pathogen immuno-genetic determinants of cryptococcosis are required. The current explosion in the development of newer immunosuppressants and biologic agents, and the emergence of infectious diseases such as Ebola disease leading to an undetermined state of chronic immunosuppression in resource-deplete areas endemic for HIV and cryptococcosis, will test us further. What the future holds and how clinicians and researchers rise to the evolving challenges is to be determined.
